# Essential Oil Composition and Biological Activity of “Pompia”, a Sardinian Citrus Ecotype

**DOI:** 10.3390/molecules24050908

**Published:** 2019-03-05

**Authors:** Guido Flamini, Laura Pistelli, Simona Nardoni, Valentina Virginia Ebani, Angela Zinnai, Francesca Mancianti, Roberta Ascrizzi, Luisa Pistelli

**Affiliations:** 1 Dipartimento di Farmacia, Università di Pisa, Via Bonanno 6, 56126 Pisa, Italy; guido.flamini@unipi.it (G.F.); luisa.pistelli@unipi.it (L.P.); 2 Centro Interdipartimentale di Ricerca “Nutraceutica e Alimentazione per la Salute” (NUTRAFOOD), Università di Pisa, Via del Borghetto 80, 56124 Pisa, Italy; laura.pistelli@unipi.it (L.P.); valentina.virginia.ebani@unipi.it (V.V.E.); angela.zinnai@unipi.it (A.Z.); francesca.mancianti@unipi.it (F.M.); 3 Dipartimento di Scienze Agrarie, Alimentari e Agro-alimentari, Università di Pisa, Via del Borghetto 80, 56124 Pisa, Italy; 4 Dipartimento di Scienze Veterinarie, Università di Pisa, Viale delle Piagge 2, 56124 Pisa, Italy; simona.nardoni@unipi.it

**Keywords:** *Citrus monstruosa*, essential oil, Pompia, antifungal activity, antibacterial activity, DPPH-scavenging activity, FRAP assay

## Abstract

Pompia is a Sardinian citrus ecotype whose botanical classification is still being debated. In the present study, the composition of Pompia peel essential oil (EO) is reported for the first time, along with that of the leaf EO, as a phytochemical contribution to the classification of this ecotype. The peel EO was tested for its antioxidant ability (with both the 2,2-diphenyl-1-picarylhydrazyl (DPPH) and ferric reducing antioxidant power (FRAP) assays). Moreover, its antimicrobial activities were tested for the first time on dermatophytes (*Microsporum canis*, *Microsporum gypseum*, and *Trichophyton mentagrophytes*), on potentially toxigenic fungi (*Fusarium solani*, *Aspergillus flavus*, and *Aspergillus niger*) as well on bacteria (*Escherichia coli*, *Staphylococcus aureus*, and *Staphylococcus pseudointermedius*). The dominant abundance of limonene in the peel EO seems to distinguish Pompia from the *Citrus* spp. to which it had previously been associated. It lacks γ-terpinene, relevant in *Citrus medica* EO. Its relative content of α- and β-pinene is lower than 0.5%, in contrast to *Citrus limon* peel EO. Pompia peel and leaf EOs did not show significant amounts of linalool and linalyl acetate, which are typically found in *Citrus aurantium*. Pompia peel EO antioxidant activity was weak, possibly because of its lack of γ-terpinene. Moreover, it did not exert any antimicrobial effects either towards the tested bacteria strains, or to dermatophytes and environmental fungi.

## 1. Introduction

“Pompia” is the popular name of a peculiar Sardinian *Citrus* ecotype, whose main area of cultivation is historically identified in the Baronia region, in the Middle-Eastern area of Sardinia island (Italy) [1]. Nowadays, however, its production has spread all over the island [2]. Its very thick albedo is used in the confectionery industry and consumed as both a candied dessert (“sa pompìa intrea”) and the main ingredient of a traditional fruit-cake (“s’aranzata”) [3].

It is a medium-sized tree (its height is lower than 3 m) with a vigorous and upright habit, with sparse branches bearing thorns and large leaves, ovate, with entire margin [4,5]. The flowers, either alone or, more frequently, grouped in inflorescences, are large, exhibiting purple-white petals [2,4,5]. The fruit represents the most peculiar morphological character of this plant: it is oblate, larger in diameter than in length; it presents a wrinkled base with a small calyx, and the peduncle is resistant to abscission [4,5]. The main features of the fruit are the exaggerated albedo (mesocarp) thickness, coupled with the rough, bumpy and very irregular peel (exocarp) appearance [1]. The immature fruit exhibits a green and hard peel, which turns yellow at full ripeness [2]. Its external irregularity has been the reason for its previous (now rejected) botanical classification with the name *Citrus medica* var. *monstruosa*: it has been hypothesized that the appearance of its peel might be due to *Eriophyes sheldoni* Ewing, 1937, also known as “citrus bud mite”, reported as a worldwide pest of citrus fruit production [6]. Camarda et al. [1] reported the absence of this mite in their samples, on which the mite *Panonychus citri* Mc Gregor was detected, instead: for the latter, however, no published literature hypothesizes a role in this fruit monstrosity.

As well as the reason for its deformities, Pompia botanical classification has been long debated and, to this day, The Plant List (http://www.theplantlist.org) and The International Plant Names Index (http://www.ipni.org) do not report any botanical name for this citrus, either as a synonym or as an unresolved name. Although the citrus presence in Sardinia was reported as early as the fifth century b.C., Pompia was first described as an individual and distinct species only in 1837, when Moris reported it as *Citrus medica monstruosa* [7]. This first classification of this citrus as a citron (*Citrus medica* L.) cultivar, though, has been rejected by Camarda et al. [1] on the basis of the evaluation of several factors, i.e., phytochemical, genetic, and morphological analyses. This work lead to the development of a new taxonomical entity defined as *Citrus limon* var. *pompia* Camarda var. *nova*, thus classifying it as a lemon cultivar, rather than a citron one [1]. The proximity of Pompia to *Citrus limon* (L.) Osbeck and *Citrus medica* L. was also reported by Mignani et al. [5]. This close relationship was evaluated on the basis of genetic markers, which showed a significant similarity of Pompia to the ‘Zagara Bianca’ lemon and ‘Etrog’ citron genotypes [5]. Petretto et al. [8], instead, evaluated the headspace volatile emissions of several citrus samples, classifying Pompia as closer to *C. aurantium*, *C. myrtifolia*, *C. sinensis*, and *C. paradisi*. Lemon and citron, on the contrary, resulted as the farthest in terms of emitted volatile organic compounds (VOCs) [8]. Curk and Luro [9] analysis of genetic *Citrus* markers classified Pompia as an hybrid of *Citrus medica* L. and *Citrus × aurantium* L. [9]. In a recent work, Deiana et al. [10] reported the VOCs emitted in the headspace of polar (ethanolic) extracts of the traditional Pompia candied flavedo (“sa pompìa intrea”). The production process did not influence the volatile emission of the peel, as limonene was found to be as abundant (over 70%) in the head space of this product as in the untreated peels analyzed by Petretto et al. [8,10].

In terms of biological activity, Fancello et al. [11] tested the hydro-distilled essential oil of Pompia leaves for its antioxidant and antimicrobial properties. Its radical scavenging ability was comparable to those of other citrus EOs, and exerted its most relevant inhibition capacity as antimicrobial on *Listeria monocytogenes* and *Staphylococcus aureus*, and on yeasts, in particular *Saccharomyces cerevisiae* and *Candida albicans*, as antifungal [11]. Ethanolic extracts of the traditional candied flavedo exhibited relevant contents of in vitro radical-scavenging phenols, such as neoeriocitrin, neohesperidin, and naringin [10].

Pompia was bestowed the Slow Food Presidium in 2004 [12]: as a peculiar and interesting ecotype, important in the Sardinian economy for its preference among consumers, Pompia represents an important biodiversity to preserve. The present study aims at aiding the botanical classification of this species through the phytochemical evaluation of the essential oil of both peels and leaves of Pompia, using a multi-organ approach. Moreover, it presents the Pompia peel essential oil antioxidant properties and the activity in inhibiting molds and bacteria growth, all of which, to the best of our knowledge, have never been investigated before.

## 2. Results

### 2.1. Phytochemical Investigation

The complete composition of the essential oils (EOs, obtained by both hydro-distillation, HD, and cold pressing, CP) of Pompia peels and leaves (extracted by hydro-distillation, HD), as well as their hydro-distillation yields are reported in Table 1. Overall, 61 compounds were identified among all the extracted EOs.

Monoterpene hydrocarbons accounted for over 80% in both the peel EOs. For the CP peels, they represented 98.38% of the EO composition, with limonene exhibiting a relative abundance of as much as 95.77%. The latter was the most abundant compound in the HD peel EO, as well, where its relative content was over 75%. Among monoterpene hydrocarbons, myrcene was the second most abundant compound, accounting for 2.12% and 1.55% in the HD and CP peel EOs, respectively. Monoterpene hydrocarbons were also abundant (42.01%) in the HD leaf EO, where limonene (28.64%) was the most abundant component in the EO, as well. In the latter sample, however, *trans*-β-ocimene showed a relevant presence (10.50%), whilst it was under 1% in both the HD and CP peel EOs.

The HD peel EO showed a larger variety of compounds, compared to the CP one: oxygenated monoterpenes, indeed, were detected in a relevant amount (16.44%), whereas they were only in traces in the CP peel EO. The HD leaf EO, instead, showed oxygenated monoterpenes as the most abundant chemical class of compounds, as they accounted for up to 53.50%. Whilst only detected in traces in the CP peel EO, geranial and neral exhibited a relevant presence in both the peel (6.16% and 4.43%, respectively) and leaf (24.44% and 18.84%, respectively) HD EOs. Geranyl and neryl acetates, only detectable in trace in the CP peel EO, accounted for circa 0.6% in the HD peel EO, and for 3.94% and 1.48%, respectively, in the HD leaf EO. Their related alcohols, instead, were lower than 1.5% in both the peel and leaf HD EOs, whilst they were not found in the CP peel EO.

Oxygenated sesquiterpenes, under 0.1% in the HD peel EO and completely absent in the CP peel EO, accounted for 3.04% in the HD leaf EO: among them, spathulenol was the most abundant (1.22%).

### 2.2. Antioxidant Activity

**Figure 1:**
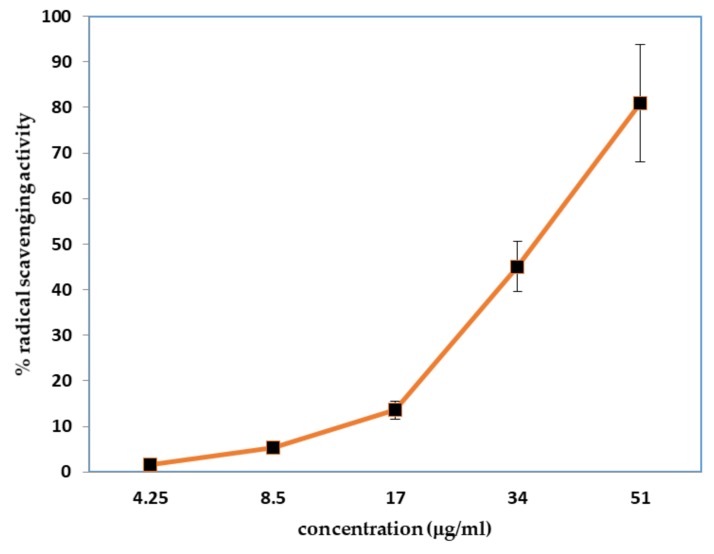
Diphenyl-1-Picarylhydrazyl (DPPH) radical scavenging activity of Pompia peel EO (*n* = 8, ± SE).

The antioxidant activity of the EO extracted by hydrodistillation from Pompia peels was determined by two different test systems: 2,2-diphenyl-1-picarylhydrazyl (DPPH) and ferric reducing antioxidant power (FRAP) assays. In the DPPH assay, the ability of the samples of interest to act as hydrogen atoms or electrons donors in the transformation of DPPH• into its reduced form DPPH—-H was investigated (Figure 1). Pompia peel EO sample presented IC_50_ values of 39.33 ± 6.1 mg/L, while ascorbic acid (used as positive control standard) had IC_50_ values of 4.70 ± 0.86 mg/L. (26.6 μM)). Some authors reported the IC_50_ for ascorbic acid in the range of 11.8–56 μM [13].

The Ferric reducing antioxidant power (FRAP) was also determined to evaluate the ability of the EO to reduce Fe^III^ through electron transfer: the activity of Pompia peel EO resulted in 1.87 ± 0.41 mg/L, while for ascorbic acid it has a value of 3364.91 ± 107.98 mg/L Fe^II^ equivalent.

### 2.3. Antifungal Activity

The EOs did not yield any antifungal activity at the tested concentrations, and an abundant mycotic growth was noticed in all the wells.

### 2.4. Antibacterial Activity

No EO activity was observed against the three selected bacterial isolates.

## 3. Discussion

The CP peels EO was dominated by limonene, which represented over 98% of the total composition. A very similar profile was reported by Camarda et al. [1] and Petretto et al. [8]: both these studies evidenced a dominant presence of limonene in the composition of the headspace emission of Pompia fruits [1,8]. The candied peel volatile emission, as well, exhibited limonene as the most abundant compound in the headspace profile [10]. No comparison can be performed with other HD peels EOs from other accessions as, to the best of our knowledge, the present work is the first report of Pompia peel hydro-distilled EO. The composition of the EO hydro-distilled from the leaves of the present study is very similar to that reported by Fancello et al.: they reported limonene as the main compound, followed by geranial, neral and *trans*-β-ocimene [11].

The rebuttal of the classification of Pompia as a citron variety seems to be confirmed by the present work, based on the significant compositional differences of the peel EO between the two species, which exhibit noteworthy qualitative differences. Several studies report the composition of *Citrus medica* L. peel EOs, extracted from specimens of different provenience and belonging to diverse genotypes: all these EOs showed a consistent limonene/γ-terpinene composition, with relevant contents of α- and β-pinene, as well [14,15,16]. Accounting for over 75% of the total composition, limonene was, instead, the defining-compound for the Pompia peels EO.

On the other hand, the proximity of Pompia to lemon proposed by Mignani et al. [5], as well as its classification as a lemon cultivar proposed by Camarda et al. [1], are only slightly more plausible. Indeed, compared to the published compositions of lemon peel EO, Pompia differed for its α- and β-pinene content: whilst these compounds were only detected in relative abundances lower than 0.5% in the analyzed Pompia peel EO, they are reported as accounting for over 1.5% and 9.0% in *Citrus lemon* (L.) Osbeck peel EOs, respectively [8,17].

The significant content of linalool in the EOs extracted from the peels of Pompia other parent, *Citrus* × *aurantium* L., represents the main compositional difference between these two species [8,18].

The antioxidant activity of Pompia peel EOs was determined for the first time, and weak activities were found in both the DPPH and FRAP assays. In the literature, only the antioxidant activity of Pompia leaf EO was evaluated [8]: it showed a weak activity in relation to the reference compounds (ascorbic acid or Trolox). *Citrus* EOs antioxidant activities have been reported for other species, such as the peel EOs from *Citrus maxima* and *Citrus sinensis*, with IC_50_ values of 8.84 and 9.45 μL/mL, respectively [19]. On the other hand, Choi et al. [20] evidenced a more relevant radical-scavenging ability of *Citrus* EOs rich in γ-terpinene and terpinolene, but in the present work γ-terpinene was not detected in Pompia peel EO, and terpinolene was only found in traces. Moreover, Choi et al. [20] concluded that limonene, although dominant in the EO compositions, had no significant role in their antioxidant activity [20].

The results dealing with the antifungal activity cannot be compared with other published data, because the effectiveness of this EO has never been checked against molds. However previous studies showed a poor activity of *Citrus limon* against the zoophilic dermatophyte *M. canis* [21]. Furthermore some *Citrus* spp yielded MIC values ranging from 2.5% to more than 5% against *A. fumigatus* [22].

Pompia peel EO inactivity towards the Gram negative *E. coli* is consistent with the results obtained with Pompia leaf EO by Fancello et al. [11], as well as other published studies, in which citrus EOs exhibited a stronger antibacterial activity on Gram positive strains [11,23,24]. The results of the present work also confirm the findings of Chubukov et al. [25]: *E. coli* resistance to limonene is due to its ability to lower the toxicity of limonene hydroperoxide through an alkyl hydroperoxide detoxifying enzyme, whose coding is a fitting advantage induced by an *ahp*C gene mutation [25]. Moreover, the peel EO showed a lower amount of oxygenated monoterpenes, which seem to be linked to the EOs antimicrobial properties [11,26].

## 4. Materials and Methods

### 4.1. Plant Material

The Pompia fruits and leaves were produced and collected by “Azienda Scuola Agraria di Siniscola—I.P.S.A.S.R.”, Località San Narciso, Siniscola (NU, Sardinia, Italy). Identification and collection were performed by Piercarlo Ferraris and Peppino Piquereddu of the agronomy school (I.P.S.A.S.R.) in Località Ghiliorro, Siniscola (NU, Sardinia, Italy) in November 2018.

### 4.2. Essential Oils Extraction

The fruit fresh peels (flavedo) were manually squeezed to obtain a cold pressed essential oil (EO): this volatile fraction was captured in a glass vial; HPLC grade *n*-hexane was added prior to GC–MS injection. The flavedo was also separated from the albedo and hydro-distilled in a Clevenger-type apparatus for 2 h: the obtained EO was diluted to 0.5% in HPLC grade *n*-hexane. The fresh leaves were roughly cut and hydro-distilled in the same apparatus and diluted in the same ratio prior to GC–MS injection.

### 4.3. Gas Chromatography–Mass Spectrometry Analyses and Peaks Identification

As reported in Ascrizzi et al. [17], the GC/EI-MS analyses were performed with a Varian CP-3800 gas chromatograph (Agilent Technologies Inc., Santa Clara, CA, USA) equipped with a non-polar DB-5 (Agilent Technologies Inc., Santa Clara, CA, USA) capillary column (length: 30 m; 0.25 mm internal diameter; film thickness 0.25 μm) and a Varian Saturn 2000 ion-trap mass detector (Agilent Technologies Inc., Santa Clara, CA, USA. The oven temperature program was set to rise from 60 °C to 240 °C at 3 °C/min. The set temperatures were as follows: injector temperature, 220 °C; transfer-line temperature, 240 °C. The carrier gas was He, at 1 mL/min flow. The injection volume was set at 1 μL. The acquisition was performed with the following parameters: full scan, with a scan range of 35–300 *m*/*z*; scan time: 1.0 s; threshold: 1 count. The identification of the constituents was based on the comparison of their retention times (t_R_) with those of pure reference samples and their linear retention indices (LRIs), which were determined relatively to the t_R_ of a series of *n*-alkanes. The detected mass spectra were compared with those listed in the commercial libraries NIST 14 and ADAMS, as well as in a homemade mass-spectral library, built up from pure substances and components of known oils, and in MS literature data [27,28,29,30,31,32].

### 4.4. Antiradical Activity by Diphenyl-1-Picarylhydrazyl (DPPH) Assay

A modified version [33] of the method proposed by Petretto and al. [8] was used to perform the radical scavenging activity. Different concentrations of EOs, in a range from 0.01 up to a maximum of 0.1 mg, were added to a solution of DPPH (100 μM in ethyl acetate), until a final volume of 1 mL was reached. The mixtures were shaken and incubated at 25 °C in the dark for 30 min. The measurement of the absorbance of the solution at 517 nm using a 1 cm quartz cuvette on a Shimadzu UV–1800 spectrophotometer was used to determine the reduction of the DPPH radical. The ascorbic calibration curve in the range 0.5–10 μg/mL was used as standard control. The percent inhibition of the DPPH radical by the samples was calculated according to the formula: % inhibition = (A_blank_ − A_sample_/A_blank_) × 100, where A_blank_ is the absorbance of the DPPH radical without the antioxidant and A_sample_ is the absorbance of the samples. Plotting a graph of the inhibition percentage against extract concentration was performed to calculate the concentration (μg/mL) of the extract providing 50% of antioxidant activities (IC_50_). All determinations were performed in triplicate.

### 4.5. Ferric Reducing Antioxidant Power (FRAP) Assay

Minor modifications [34] to the ferric reducing antioxidant power (FRAP) method were used to assess a second determination of the antioxidant power of Pompia peel EO. The freshly prepared working solution always contained 7.5 mM acetate buffer, pH 3.6, 0.1 mM tripyridyltriazine (TPTZ), and 0.05 mM FeCl_3_·6H_2_O. At low pH, the tripyridyltriazine (Fe^III^-TPTZ) complex is reduced to the ferrous form (Fe^II^): the reduced complex (Fe^II^-TPTZ) exhibits an intensive blue color that can be monitored spectrophotometrically at 593 nm. Aqueous solution of known Fe^II^ concentration was used for calibration (in a range of 100–1000 μmol/L) as external standard reference.

### 4.6. Antifungal Activity

Antifungal activity of the EOs was checked against both dermatophytes and potentially toxigenic fungi. In detail, a clinical isolate of *M. canis*, *M. gypseum*, *T. mentagrophytes* (from feline source), respectively, and *F. solani*, *A. flavus* and *A. niger* isolated from the environment were used for antifungal testing. The determination was carried out by a microdilution test, performed as recommended by the Clinical and Laboratory Standards Institute (CLSI) M38-A2 for molds (2008) [35], with slight modification, starting from a 5% dilution. Five percent, 2.5%, 2%, 1.5% and 1% dilutions in semisolid medium were achieved. All the assays were performed in triplicate.

### 4.7. Antibacterial Activity

To verify the antibacterial activity, the EOs were tested against Gram negative and Gram positive bacteria. In particular, the analyses were carried out employing *Escherichia coli*, *Staphylococcus aureus*, and *Staphylococcus pseudointermedius* strains, previously isolated from clinical canine specimens.

Minimum inhibitory concentration (MIC) was determined for all EOs with the broth microdilution method, starting from a dilution of 10% (*v*/*v*) and following the guidelines of the Clinical and Laboratory Standards Institute (CLSI) (1990) [36], with some modifications as previously reported [37]. All the assays were performed in triplicate.

## 5. Conclusions

From a phytochemical point of view, Pompia showed peculiar EO compositions for both its peel and leaves, which seem to indicate its individuality as a species of its own, rather than its classification as a genotype or variety of lemon, citron, or sour orange.

The Pompia peel EO exhibited a weak antioxidant activity, on both the DPPH and the FRAP assays. Moreover, it exerted no antimicrobial activity, either on the tested Gram positive and negative bacteria strains, or on the evaluated dermatophytes and environmental fungi.

## Figures and Tables

**Table 1:** Complete composition and hydro-distillation yield of the *Citrus limon* var. *pompia* Camarda var. *nova* essential oils extracted from the peels (by hydro-distillation, HD, and cold pressing, CP) and from the hydro-distilled leaves.

	Constituents	l.r.i. ^a^	Relative Abundance (%) ± SD		
			HD Peel EO	CP Peel EO	HD Leaf EO
1	α-thujene	931	Tr ^b^	- ^c^	-
2	α-pinene	941	0.43 ± 0.00	0.51 ± 0.04	tr
3	camphene	954	tr	-	-
4	sabinene	976	0.14 ± 0.00	0.07 ± 0.08	0.28 ± 0.01
5	β-pinene	982	tr	-	0.19 ± 0.00
6	6-methyl-5-hepten-2-one	985	tr	-	tr
7	myrcene	993	2.12 ± 0.01	1.55 ± 0.04	0.91 ± 0.02
8	α-phellandrene	1005	tr	tr	tr
9	δ-3-carene	1011	tr	-	0.90 ± 0.05
10	α-terpinene	1018	tr	-	-
11	*p*-cymene	1027	-	-	tr
12	limonene	1032	77.44 ± 0.58	95.77 ± 0.30	28.64 ± 1.24
13	*cis*-β-ocimene	1042	-	-	0.46 ± 0.04
14	*trans*-β-ocimene	1052	0.99 ± 0.01	0.50 ± 0.13	10.50 ± 0.12
15	*iso*terpinolene	1087	-	-	tr
16	terpinolene	1088	tr	-	0.16 ± 0.02
17	linalool	1101	1.13 ± 0.06	tr	0.56 ± 0.02
18	*trans*-*p*-mentha-2,8-dien-1-ol	1121	tr	-	-
19	*trans*-limonene oxide	1141	tr	-	tr
20	camphor	1143	tr	-	-
21	citronellal	1155	0.33 ± 0.00	-	1.27 ± 0.16
22	*iso*neral	1171	tr	-	0.37 ± 0.14
23	4-terpineol	1178	tr	-	-
24	*iso*geranial	1184	0.14 ± 0.01	-	0.44 ± 0.04
25	α-terpineol	1189	0.35 ± 0.01	-	0.14 ± 0.02
26	decanal	1204	-	-	tr
27	*trans*-carveol	1218	tr	-	-
28	nerol	1230	1.24 ± 0.04	-	1.49 ± 0.09
29	neral	1240	4.43 ± 0.14	tr	18.84 ± 0.36
30	geraniol	1257	1.46 ± 0.01	-	0.57 ± 0.02
31	geranial	1271	6.16 ± 0.13	tr	24.44 ± 1.59
32	methyl geranate	1325	tr	-	-
33	citronellyl acetate	1350	tr	-	tr
34	neryl acetate	1366	0.61 ± 0.03	tr	1.48 ± 0.05
35	geranyl acetate	1385	0.62 ± 0.02	-	3.94 ± 0.18
36	*cis*-α-bergamotene	1416	tr	tr	-
37	β-caryophyllene	1420	0.42 ± 0.01	0.34 ± 0.04	0.76 ± 0.03
38	β-copaene	1429	tr	-	-
39	*trans*-α-bergamotene	1438	0.59 ± 0.04	0.55 ± 0.06	tr
40	α-humulene	1456	tr	tr	0.11 ± 0.01
41	*trans*-β-farnesene	1460	0.16 ± 0.01	-	-
42	9-*epi*-*trans*-caryophyllene	1467	-	tr	-
43	germacrene D	1478	tr	-	-
44	γ-curcumene	1480	tr	-	-
45	valencene	1492	0.13 ± 0.01	-	tr
46	bicyclogermacrene	1495	0.27 ± 0.02	0.26 ± 0.01	0.45 ± 0.01
47	*cis*-α-bisabolene	1504	tr	tr	-
48	β-bisabolene	1509	0.89 ± 0.06	0.48 ± 0.11	0.13 ± 0.01
49	δ-cadinene	1524	tr	-	tr
50	*trans*-α-bisabolene	1531	tr	-	-
51	*trans*-nerolidol	1565	tr	-	0.40 ± 0.02
52	germacrene D-4-ol	1575	tr	-	tr
53	spathulenol	1576	-	-	1.22 ± 0.11
54	caryophyllene oxide	1581	-	-	0.73 ± 0.02
55	β-oplopenone	1606	-	-	tr
56	*epoxy*-*allo*aromadendrene	1639	-	-	tr
57	*epi*-α-cadinol	1640	-	-	0.21 ± 0.06
58	α-cadinol	1654	-	-	0.39 ± 0.01
59	valerianol	1656	tr	-	-
60	β-bisabolol	1672	-	-	0.10 ± 0.14
61	*epi*-α-bisabolol	1686	tr	-	tr
	Monoterpene hydrocarbons		81.12 ± 0.59	98.38 ± 0.00	42.01 ± 1.49
	Oxygenated monoterpenes		16.44 ± 0.44	tr	53.50 ± 1.45
	Sesquiterpene hydrocarbons		2.45 ± 0.15	1.62 ± 0.00	1.44 ± 0.04
	Oxygenated sesquiterpenes		tr	-	3.04 ± 0.07
	Non-terpene derivatives		tr	-	tr
	Total identified (%)		100.00 ± 0.00	100.00 ± 0.00	99.98 ± 0.01
	Extraction yield (% *w*/*w*)		0.3	n.a.	<0.1%

^a^ Linear retention indices on a DB5 column; ^b^ Traces, <0.1%; ^c^ Not detected.

## References

[1] Camarda I., Mazzola P., Brunu A., Fenu G., Lombardo G., Palla F. (2013). Un agrume nella storia della Sardegna: *Citrus limon* var. *pompia* Camarda var. *nova*. Quad. di Bot. Ambient. e Appl..

[2] D’Aquino S., Fronteddu F., Usai M., Palma A. (2005). Qualitative and physiological properties of ‘Pompia’, a citron-like fruit. Plant Genet. Resour. Newsl. IPGRI.

[3] Sa pompia: Un particolare frutto da cui si ottiene un dolce tipico di Siniscola. http://www.siniscolaonline.it/pompia.htm.

[4] Chessa I., Mulas M., Pala M., Agabbio M. (1994). Gli Agrumi. Le vecchie varietà di Sardegna. Patrimonio genetico di specie arboree da frutto.

[5] Mignani I., Mulas M., Mantegazza R., Lovigu N., Spada A., Nicolosi E. (2015). Daniele Bassi Characterization by Molecular Markers of ‘Pompia’, a Natural Citrus Hybrid Cultivated in Sardinia. Acta Hortic..

[6] INRA Encyclopédie des ravageurs européens: *Eriophyes sheldoni* (Ewing). http://www7.inra.fr/hyppz/RAVAGEUR/6erishe.htm.

[7] Moris G.G. (1837). Flora sardoa 1.

[8] Petretto G.L., Sarais G., Maldini M.T., Foddai M., Tirillini B., Rourke J.P., Chessa M., Pintore G. (2016). Citrus monstruosa Discrimination among Several *Citrus* Species by Multivariate Analysis of Volatiles: A Metabolomic Approach. J. Food Process. Preserv..

[9] Curk F., Luro F. (2018). Classificazione ed Evoluzione Degli Agrumi. Oral communication.

[10] Deiana M., Montoro P., Jerković I., Atzeri A., Marijanović Z., Serreli G., Piacente S., Tuberoso C.I.G. (2018). First characterization of Pompia intrea candied fruit: The headspace chemical profile, polar extract composition and its biological activities. Food Res. Int..

[11] Fancello F., Petretto G.L., Zara S., Sanna M.L., Addis R., Maldini M., Foddai M., Rourke J.P., Chessa M., Pintore G. (2016). Chemical characterization, antioxidant capacity and antimicrobial activity against food related microorganisms of *Citrus limon* var. *pompia* leaf essential oil. LWT Food Sci. Technol..

[12] Pompia. https://www.fondazioneslowfood.com/en/slow-food-presidia/pompia/.

[13] Sharma O.P., Bhat T.K. (2009). DPPH antioxidant assay revisited. Food Chem..

[14] Lota M.-L., de Rocca Serra D., Tomi F., Bessiere J.-M., Casanova J. (1999). Chemical composition of peel and leaf essential oils of *Citrus medica* L. and *C. limonimedica* Lush. Flavour Fragr. J..

[15] Venturini N., Curk F., Desjobert J.-M., Karp D., Costa J., Paolini J. (2010). Chemotaxonomic Investigations of Peel and Petitgrain Essential Oils from 17 Citron Cultivars. Chem. Biodivers..

[16] Menichini F., Tundis R., Bonesi M., de Cindio B., Loizzo M.R., Conforti F., Statti G.A., Menabeni R., Bettini R., Menichini F. (2011). Chemical composition and bioactivity of *Citrus medica* L. cv. *Diamante* essential oil obtained by hydrodistillation, cold-pressing and supercritical carbon dioxide extraction. Nat. Prod. Res..

[17] Ascrizzi R., Taglieri I., Sgherri C., Flamini G., Macaluso M., Sanmartin C., Venturi F., Quartacci M., Pistelli L., Zinnai A. (2018). Nutraceutical Oils Produced by Olives and Citrus Peel of Tuscany Varieties as Sources of Functional Ingredients. Molecules.

[18] Trabelsi D., Hamdane A.M., Said M.B., Abdrrabba M. (2016). Chemical Composition and Antifungal Activity of Essential Oils from Flowers, Leaves and Peels of Tunisian *Citrus aurantium* Against *Penicillium digitatum* and *Penicillium italicum*. J. Essent. Oil Bear. Plants.

[19] Singh P., Shukla R., Prakash B., Kumar A., Singh S., Mishra P.K., Dubey N.K. (2010). Chemical profile, antifungal, antiaflatoxigenic and antioxidant activity of *Citrus maxima* Burm. and *Citrus sinensis* (L.) Osbeck essential oils and their cyclic monoterpene, dl-limonene. Food Chem. Toxicol..

[20] Choi H.-S., Song H.S., Ukeda H., Sawamura M. (2000). Radical-Scavenging Activities of Citrus Essential Oils and Their Components: Detection Using 1,1-Diphenyl-2-picrylhydrazyl. J. Agric. Food Chem..

[21] Mugnaini L., Nardoni S., Pinto L., Pistelli L., Leonardi M., Pisseri F., Mancianti F. (2012). In vitro and in vivo antifungal activity of some essential oils against feline isolates of *Microsporum canis*. J. Mycol. Med..

[22] Ebani V., Najar B., Bertelloni F., Pistelli L., Mancianti F., Nardoni S., Ebani V.V., Najar B., Bertelloni F., Pistelli L. (2018). Chemical Composition and In Vitro Antimicrobial Efficacy of Sixteen Essential Oils against *Escherichia coli* and *Aspergillus fumigatus* Isolated from Poultry. Vet. Sci..

[23] Burt S. (2004). Essential oils: Their antibacterial properties and potential applications in foods—A review. Int. J. Food Microbiol..

[24] Ruiz B., Flotats X. (2014). Citrus essential oils and their influence on the anaerobic digestion process: An overview. Waste Manag..

[25] Chubukov V., Mingardon F., Schackwitz W., Baidoo E.E.K., Alonso-Gutierrez J., Hu Q., Lee T.S., Keasling J.D., Mukhopadhyay A. (2015). Acute Limonene Toxicity in *Escherichia coli* Is Caused by Limonene Hydroperoxide and Alleviated by a Point Mutation in Alkyl Hydroperoxidase AhpC. Appl. Environ. Microbiol..

[26] Settanni L., Palazzolo E., Guarrasi V., Aleo A., Mammina C., Moschetti G., Germanà M.A. (2012). Inhibition of foodborne pathogen bacteria by essential oils extracted from citrus fruits cultivated in Sicily. Food Control.

[27] Masada Y. (1976). Analysis of Essential Oils by Gas Chromatography and Mass Spectrometry.

[28] Swigar A.A., Silverstein R.M. (1981). Monoterpenes.

[29] Jennings W., Shibamoto T. (1982). Qualitative Analysis of Flavor and Fragrance Volatiles by Glass Capillary Gas Chromatography.

[30] Davies N.W. (1990). Gas chromatographic retention indices of monoterpenes and sesquiterpenes on Methyl Silicon and Carbowax 20M phases. J. Chromatogr. A.

[31] Adams R.P. (1995). Identification of eSsential Oil Components by Gas Chromatography/Quadrupole Mass Spectroscopy.

[32] Adams R.P., Zanoni T.A., Lara A., Barrero A.F., Cool L.G. (1997). Comparisons among *Cupressus arizonica* Greene, *C. benthamii* Endl., *C. lindleyi* Klotz, ex Endl. and *C. lusitanica* Mill, using Leaf Essential Oils and DNA Fingerprinting. J. Essent. Oil Res..

[33] Petretto G.L., Maldini M., Addis R., Chessa M., Foddai M., Rourke J.P., Pintore G. (2016). Variability of chemical composition and antioxidant activity of essential oils between *Myrtus communis* var. *Leucocarpa* DC and var. *Melanocarpa* DC. Food Chem..

[34] Szőllősi R., Szôllôsi Varga I. (2002). Total antioxidant power in some species of Labiatae (Adaptation of FRAP method). Acta Biol. Szeged..

[35] (2008). CLSI Reference Method for Broth Dilution Antifungal Susceptibility Testing of Filamentous Fungi.

[36] (1990). CLSI Methods for Dilution Antimicrobial Susceptibility Tests for Bacteria that Grow Aerobically.

[37] Ebani V.V., Nardoni S., Bertelloni F., Giovanelli S., Rocchigiani G., Pistelli L., Mancianti F. (2016). Antibacterial and antifungal activity of essential oils against some pathogenic bacteria and yeasts shed from poultry. Flavour Fragr. J..

